# Hierarchical Structure in Sequence Processing: How to Measure It and Determine Its Neural Implementation

**DOI:** 10.1111/tops.12442

**Published:** 2019-07-30

**Authors:** Julia Uddén, Mauricio de Jesus Dias Martins, Willem Zuidema, W. Tecumseh Fitch

**Affiliations:** ^1^ Department of Psychology, Department of Linguistics Stockholm University; ^2^ Swedish Collegium for Advanced Study (SCAS); ^3^ Berlin School of Mind and Brain Humboldt Universität zu Berlin; ^4^ Max Planck Institute for Human Cognitive and Brain Sciences; ^5^ Clinic for Cognitive Neurology University Hospital Leipzig; ^6^ Institute for Logic, Language and Computation University of Amsterdam; ^7^ Department of Cognitive Biology, Faculty of Life Sciences University of Vienna

**Keywords:** Hierarchical structure, Sequence processing, Nested grouping, Neural signatures

## Abstract

In many domains of human cognition, hierarchically structured representations are thought to play a key role. In this paper, we start with some foundational definitions of key phenomena like “sequence” and “hierarchy," and then outline potential signatures of hierarchical structure that can be observed in behavioral and neuroimaging data. Appropriate behavioral methods include classic ones from psycholinguistics along with some from the more recent artificial grammar learning and sentence processing literature. We then turn to neuroimaging evidence for hierarchical structure with a focus on the functional MRI literature. We conclude that, although a broad consensus exists about a role for a neural circuit incorporating the inferior frontal gyrus, the superior temporal sulcus, and the arcuate fasciculus, considerable uncertainty remains about the precise computational function(s) of this circuitry. An explicit theoretical framework, combined with an empirical approach focusing on distinguishing between plausible alternative hypotheses, will be necessary for further progress.

## Abstract 2

Spoken language consists of a linear sequence of units, from which the existence of particular underlying hierarchical processing mechanisms is inferred. Uddén et al. use graph theory to provide a framework for describing the possible structural relationships that may underlie a linear output sequence. Being more explicit in defining different structures can help identifying and testing for such structures in AGL experiments, as well as help showing how behavioral and neuroimaging data reveals signatures of hierarchical processing in humans.

## 1 The challenge of hierarchy

Since the cognitive revolution, the cognitive and neurosciences have sought an account of perception, motor, and higher cognitive faculties such as language and memory in terms of specific representations*.* In several cognitive domains, including most prominently language, a seminal suggestion (Chomsky, 1957; Lashley, 1951; Simon, 1962) is that the human mind creates *hierarchical* representations, even when the sensory input is sequentially presented (or the output is a sequence of actions).

For most linguists, the hierarchical nature of linguistic representations is self‐evident, and most explicit theories of language processing take hierarchical abilities as a given, as do several theories of musical structure (Fitch, 2013; Lerdahl & Jackendoff, 1983). However, empirically demonstrating the existence of hierarchical structure in cognition, particularly outside of language, remains a challenge for at least two reasons. One is terminological: because scholars use the term “hierarchical” in different ways, a valid test for one conception of hierarchy may not apply to another. The second is more interesting and substantial: Our lack of direct access to cognitive structures means that certain types of hierarchies cannot be distinguished, empirically, from other structures (e.g., sequences).

Current controversy in neurolinguistics illustrates this point. Distinct theories of syntax posit different hierarchical operations, leading researchers to analyze the neural basis of syntax in terms of “move” (Caplan, 1987), “merge” (Berwick et al., 2013), or “unify” (Hagoort, 2005). Additional hierarchy‐building operators are also available (e.g., “adjoin” in tree adjoining grammar; Joshi, 2003). However, it is a true challenge, if possible, to empirically distinguish among such fine theoretical distinctions due to the “granularity mismatch” problem (Embick & Poeppel, 2015). Future progress will require a unified perspective broad enough to capture what these syntactic operators have in common, but specific enough to distinguish hierarchical from sequential processing.

In this paper, we thus begin with unambiguous, explicit definitions of key concepts for the following discussion, especially “hierarchy," “sequence,"and “tree” (see also Fitch, 2013; Zuidema, Hupkes, Wiggins, Scharff, & Rohrmeier, 2018). This provides an explicit framework for the following review, which focuses on how to empirically distinguish between hierarchical and sequential processing in different domains. Our goal is not to develop a new theory of syntax or hierarchy, but rather to use well‐established terminology from mathematics (especially graph theory) to clarify and sharpen our subsequent data‐focused review. Only from such a general perspective will it be possible to determine whether the behavioral and neural signatures of hierarchy differ between domains.

With these clarifications in hand, we then turn to our main focus: critically reviewing possible empirical indicators of hierarchical structure and/or processing that have been proposed, beginning with behavioral data and then turning to neuroimaging data. We argue that despite considerable controversy concerning terminology and theory, there is consistent converging evidence that a specific frontotemporal network is involved in hierarchy building, and this network is similarly activated by hierarchical processing in different domains (especially music and language).

## 2 Definitions

Our goal is to formulate a general but explicit classification of different hierarchical and non‐hierarchical structures, allowing comparisons of linguistic hierarchical structure and processing with that in other domains such as music or social cognition. Given this goal, we must avoid formulations that prematurely embody or entail language‐ or music‐specific constructs (e.g., c‐command or musical key) while allowing space for those constructs as special cases. To achieve this, we adopt the overarching terminology of graph theory, in which such fundamental notions as “sequence," “tree," and “network” can be explicitly defined. Graph theory is clear, well‐developed, widely known, and widely used in computer science, as well as neuroscience (Bullmore & Sporns, 2009). Although other formulations are possible, particularly for domain‐specific applications, they lack the combination of generality and clarity that we aim for here. For example, there are set‐theoretic models of syntax, providing an alternative formulation of hierarchical containment relations via subsets, as well as model theoretic accounts and vector‐space models (see, e.g., Pullum & Scholz, 2001; Repplinger, Beinborn, & Zuidema, 2018). The difficulty is that sets are by definition unordered, thus excluding the core notion of “sequence.” Furthermore, mathematical sets cannot contain duplicates (while graphs can) and thus are ill‐suited as representations of sentences with repeated words or melodies with repeated notes.

### 2.1 Hierarchical and sequential structure

The notion of hierarchical structure that we are interested in contrasts with sequential structure. How can we define these terms formally? We examine these concepts from the perspective of graph theory and computing theory (see Harel & Feldman, 1987; Wilson, 1996 for accessible introductions). We will consider structures over a collection of discrete, categorical *items*: These could be collections of words, notes, syllables, phones, primitive actions, or any other entities that are represented by the brain, but also cognitive categories that encompass multiple items, such as the categories of birds, nouns, or nasal consonants.

**Figure 1:**
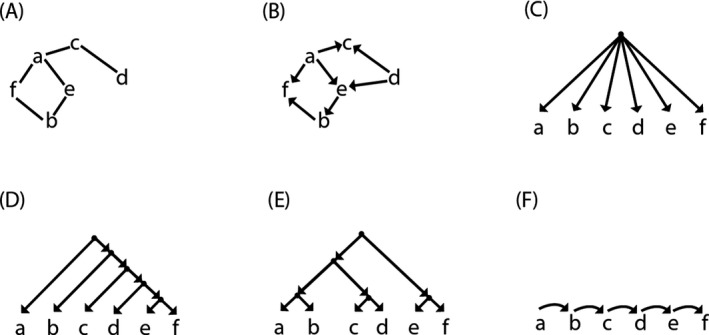
(A) Connected graph; (B) directed acyclic graph (DAG); (C) rooted DAG; (D) right‐branching tree; (E) multiply nested tree; (F) sequence. Non‐terminal nodes in C, D, and E are represented as black dots; other items as letters. The crucial difference between hierarchies (C, D, and E) and sequences (F), both rooted DAGs, is that in the former, at least one node has more than one child, which implies that hierarchies have more than one terminal. Although it is conventional to represent terminal nodes as ordered from left to right, these terminal nodes can be either unordered or ordered using some supplemental enumeration method (e.g., alphabetic).

A *graph* is a mathematical structure composed of *nodes* representing items and *edges* connecting nodes. Edges represent arbitrary relationships between these items (such as “close to,” “resembles,” “implies”, etc.). There is no further limitation on graphs, though we will confine ourselves here to *connected* graphs, where every item is linked to the group via at least one link (Fig. 1A). An intuitive example of a graph would be the nations of the world, with the distances to their neighbors indicated by the edges.

A graph is a very general notion; restrictions on the “graph” definition create subtypes, such as directed acyclic graphs (DAGs), where the edges have a directionality (Fig. 1B), pointing from *parent* node to *child* node. "Acyclic" means that there are no circles or loops in this structure, implying that no node can (indirectly) dominate itself. In DAGs, a terminal may have more than one parent node, but the graph nonetheless remains acyclic. *Terminal nodes* are connected to only their parent and have no dependent nodes (they have "one end free"): Terminals often represent explicit, perceptibly expressed items (e.g., numbers, words, musical notes, individuals, etc.) but sometimes also “null elements," “traces," etc. (in linguistics) or silent rests (in music). *Non‐terminal nodes* are connected with at least one child, and they can be either perceptually expressed (as in Fig. 1F) or not (as in Fig. 1E). When these nodes are not explicitly represented and need to be inferred by the listener/viewer, they are often termed *internal nodes*.

An important subtype is the “rooted” graph, a graph which has a *root node* (Fig. 1C). The notion of a root node is intuitive: There is some single node from which the entire rest of the graph can be said to emanate, meaning that this node is the (indirect) parent of all others.

A simple example of a rooted DAG is a *sequence,* which is a group of items that is accessed in a specified order (e.g., the alphabet [a,b,c, …, z]). In sequences, each node has exactly one child (except for the terminal, which has none) and one parent (except for the *root*) (Fig. 1F), thus enforcing an obligatory reading order. Accordingly, sequences have a single terminal. These limitations do not apply to hierarchies.

*Hierarchy* entails a more complex rooted DAG in which at least one node has more than one child, and every node has exactly one parent (except for the root). Since a parent with two children implies “branching” of the directed graph, hierarchies are commonly called *trees*.

Both sequences and trees are rooted DAGs, in which items are ordered or ranked along a “root‐to‐terminal” axis. In the case of sequences, there is only one path from root‐to‐terminal (the final element). In the case of hierarchies, “branching” implies several root‐to‐terminal paths and, therefore, more than one terminal. This crucial difference endows hierarchies with an additional group of items—the set of terminals = {terminal1, terminal2, …}—which is unordered. This unordered set creates a secondary representational dimension along a “terminal‐to‐terminal” axis, which can acquire any arbitrary perceptual organization (spatial or temporal), independent of the root‐to‐terminal order.

We can now use these structural definitions to define our central concepts:

***Sequential structure:*** a rooted DAG in which no node has more than one child, thus being limited to a single order along the root‐to‐terminal axis and possessing a single terminal.

***Hierarchical structure:*** a rooted DAG in which at least one node has more than one child, thus forming a branching tree. In this structure, items are ordered along a root‐to‐terminal axis. In addition, due to branching, there is more than one terminal. Unless specified by some secondary method, the set of terminals is unordered along the terminal‐to‐terminal axis.

This distinction allows us to frame a central aspect of trees in human cognition: They frequently embody both hierarchical *and* sequential structure. In language, utterances contain words in a sequence, while musical melodies have notes in sequence. Perceptually, words or notes are expressed through time in a sequential manner. At the same time, syntactic relations between these elements typically implies hierarchical structure which cannot be fully represented by the perceptually explicit sequential structure. This means that a listener processing a string of items (where only the sequential structure is explicit) must infer the internal nodes that determine the hierarchical structure, of which words or notes are only the set of terminals. Although clues to this hierarchical structure may be present, including speech prosody, embedding markers like “that," or musical phrasing, these do not fully specify the structure. Thus, trees exist in the mind of the beholder, not in the perceptual stimulus itself. A key desideratum in understanding hierarchical cognition is thus understanding how and why hierarchical structures can be reliably output as sequences (cf. Kayne, 1994), and how those sequences converted (“parsed”) back into hierarchical structures. The existence of additional hierarchical representations that perceivers impose or “project” onto a sequentially presented stimulus affords several key signatures of hierarchy, discussed below.

Hierarchical representations of linguistic structure are central in all major linguistic theories, including theories of phonological structure, theories of morphological structure, theories of sentence‐level semantics, theories of dialogue and discourse structure, and both phrase‐structure and dependency‐structure‐based theories of syntax. Trees are the simplest graphs that can account for argument structure (“who does what to whom”) and the productivity of language. However, they are not complex enough to account for certain syntactic phenomena such as pronouns and anaphora, or sentences such as “Mary pretends not to hear” (where Mary is the subject of both “pretend” and “hear”). Linguists would argue that such phenomena necessitate more complex graphs than trees, as do more unusual—and controversial—phonological phenomena such as ambisyllabicity, where the same consonant is “owned” by two different syllables. Hierarchical structure is also assumed in many theories of musical structure (Lerdahl & Jackendoff, 1983; Rohrmeier, 2011), although empirical demonstrations distinguishing hierarchical from sequential structure turn out to be challenging. The difficulties stem from the fact that, as mentioned above, in many cognitive domains including language, music, and action, tree structures are “serialized” for performance, so that each hierarchical terminal (word, note, action, etc.) is perceptually expressed in a specific sequential order.

The central difficulty in clearly distinguishing hierarchical from sequential structure is illustrated by Fig. 1C, 1, and 1, which shows three examples of structures that are unambiguously hierarchical, theoretically, but if read out serially (from left to right) are very difficult to distinguish from purely sequential structures.

We will focus on sequentially presented stimuli, discussing signatures of hierarchical structure (i.e., representation) and generation processes separately. An overview of the methods is present in Table 1.

**Table 1:** Overview of methods. Some methods can formally establish the presence of hierarchical structure, while others rather are simply compatible with the presence of such structure (see text)

Distance methods
Hierarchical distance shorter than sequential distance
Levelt’s analysis of similarity/relatedness
Automatic hierarchical clustering methods
Presence of long distance dependencies
Generalization and error‐based methods
Hierarchical generalization and foils
Structural priming
Deletions and insertions

### 2.2 Signatures of hierarchical structures in representation: Distance methods

One class of approaches for demonstrating the cognitive reality of hierarchy distinguishes between “hierarchical distance,” which is the number of intervening superior nodes in the path from one terminal to another, from “sequential distance,” which is simply how many intervening *terminals* we see in the sequential output. This distinction lies at the heart of many empirical indicators of hierarchical structure.

This method cannot, however, be applied to all hierarchies. For instance, in Fig. 1C all terminals are hierarchically next‐door neighbors, even though sequentially at different distances. Only if we had unambiguous measures of hierarchical and sequential distance could we demonstrate that the terminals are hierarchically arranged. In the “right branching” tree, Fig. 1D, the difficulty is the opposite: Sequential and hierarchical distances are perfectly correlated. Terminal ‘a’ is just as far, hierarchically, from terminal ‘e’ as it is sequentially. In both cases, it will be challenging to evaluate the hierarchical structure empirically using distance methods. Fig. 1E shows the type of tree that supports unambiguous attribution of hierarchy. Here, a multiply nested tree has terminal pairs where sequential and hierarchical distances are clearly different: Although the sequential distance from c to d is the same as d to e, hierarchically c and d are neighbors while d and e are four nodes apart.

In natural language, the sequential/hierarchical distance distinction provides the clearest demonstration of hierarchy, using semantic interpretation. Given the sentence “the boy who fed the dog chased the girl," we can ask the semantically based question “who chased the girl." The answer is “the boy”: Although “the dog” is closer to “chased” in the sequence, its hierarchical distance is longer than the hierarchical distance from “boy” to “chased." This and many other phenomena in syntax make language a domain where the presence of tree structure is practically undisputed (although its pervasiveness has recently been questioned (Frank, Bod, & Christiansen, 2012)).

Levelt (1970a,1970b) developed a behavioral paradigm to test the psychological reality of hierarchical structure in a sentence processing experiment, based on Johnson (1967). Levelt first investigated how the probability of identification/recall of each word in a sentence presented in noise depended on the identification/recall of other words in that sentence (Levelt, 1970a). High conditional probabilities suggest that a cluster (a “subgroup” in our terms) was formed between these words. Additionally, participants ranked similarities between all possible pairs of three randomly selected words. High similarity rankings between two words suggest these words form a subgroup. Levelt then used each hierarchical structure derived from these data as a model to generate predictions then tested on the data, creating a measure of fit of the best hierarchical model. A very good fit implies the psychological reality of the hierarchical structured analysis. In Levelt’s case, only about 5% of the model predictions failed to show up in the data (Levelt, 1970a); he thus concluded that hierarchical structure was indeed present. Outside the domain of language, analysis of response times across sequences of keypresses during motor learning have also been used to demonstrate patterns consistent with the representation of motor clusters, which cannot be explained by simple sequential associations (Hunt & Aslin, 2001; Verway et al., 2011; Verway & Wright, 2014).

Demonstration of long‐distance dependencies, where the interpretation of one part of the sequence depends on another, distant, part, is also indicative of hierarchical structure (like the “boy chased” example above). To establish that a long‐distance dependency is present, we can generate stimuli using a suitable artificial grammar and then test if participants parse the stimuli hierarchically using the similarity/relatedness method. Long‐distance dependencies require memory, which can also be investigated (see Section 3.1). This permits investigation of sequences with multiple long‐distance dependencies, whether crossed or nested (for crossing dependencies see, e.g., Udden, Ingvar, Hagoort, & Petersson, 2017). Successful processing of nested long‐distance dependencies in classical music has also been demonstrated, using a violation paradigm (Koelsch et al, 2013). These authors, however, point out that it is still unclear whether multiple (more than one) simultaneous embedded dependencies are processed in music.

These methods have been applied in domains where semantically based diagnostics do not apply, such as prosody. Certain prosodic phenomena, such as phrase‐final lengthening, provide indications of phrase structure, and if these are nested are consistent with a hierarchical interpretation (Morgan, 1996).

### 2.3 Generalization and error‐based methods

Convincing evidence for the presence of hierarchical structure is also provided by hierarchical generalization, when a set of terminals can be flexibly rearranged in a way that obeys a posited hierarchical structure (e.g., “the girl who fed the boy chased the dog," “the dog who fed the girl chased the boy," etc.) without generating ill‐formed alternatives (“the dog fed chased the boy the girl”). In an artificial grammar learning (AGL) experiment, for instance, we can investigate whether a participant generalizes to new sequence exemplars following a hierarchical grammar, while rejecting sequences violating the grammar (but including the same collection of terminals) as non‐grammatical. To be convincing, such experiments should evaluate whether participants exposed to training stimuli generated by hierarchical rules generalize to new exemplars of different lengths and reject carefully‐selected foils (cf. Fitch & Friederici, 2012). The approach of testing the ability to generate well‐formed hierarchical structures by acquiring the appropriate generative rules and applying them beyond the given perceptual stimuli has also been successfully used in the visual‐spatial, motor, and tonal domains (Jiang et al., 2018; Martins et al., 2019, 2014, 2017).

Another behavioral method is termed *structural priming* (cf. Branigan & Pickering, 2017). Structural priming experiments can establish that, for example, sentence structure is primed, rather than specific terminals, by replacing terminals from the “prime” sequence in the “target” sequence. Recognition (or production) of the target sequence is then facilitated by recent exposure to the prime sequence. Priming effects are typically quantified by decreased reaction times or decreased neural activity. Structural priming does not, however, provide definitive proof of hierarchical structure: A priming effect only shows that some kind of structure was primed, but it does not necessarily distinguish hierarchical from sequential structure (not until this difference is specifically addressed).

Finally, in sequences, a node is only connected to other adjacent nodes. Thus, deletion of any node (except for the first and last) should hinder generation of the sequence. If participants halt when facing deletions or insertions, this suggests sequential rather than hierarchical structure. However, this method also does not provide a definitive proof, because hesitations or increased error rates are the probable observable outcomes of such a halt, and these effects might also be predicted (albeit to a lesser extent) by hierarchical structure.

### 2.4 Signatures of hierarchical structures in generating processes

Online behavioral and neural data can be analyzed to test the psychological reality of the processes that generate hierarchical structures, if these processes are made computationally explicit by means of a processing model (e.g., the grouping, ordering and nested grouping/hierarchical branching processes discussed above). A processing model, including, for example, nested grouping, must also specify that increased load on some part of the process will lead to increased effort (“effort” in this context does not imply deliberate thought processes). For behavioral data, online measures such as reading/response time, or performance under dual task conditions, provide metrics to measure effort. Additional online methods for measuring effort include eye‐tracking fixation time data, or neural measures including deflections of particular ERP‐responses, oscillatory MEG responses, electrocorticography (ECoG) data, or fMRI BOLD‐responses. An underlying hierarchical structure is suggested when increased load in a putative nested grouping process correlates with increased effort as measured by such behavioral and neural signals.

A seminal example of this approach is an fMRI‐study by Pallier et al. (2011), which presented word groups of different sizes, varying from 2 to 12 words, but always in 12‐word sequences (thus one to six groups per sequence). Assuming larger constituent sizes require increased activity in group‐generating processes, any neural signal that parametrically increases with constituent size is potentially diagnostic of hierarchical structure building. The location of activity can furthermore indicate where such computational processes are implemented. Using a Jabberwocky (nonce word) condition, where content words are replaced with non‐words to control for semantic processes, Pallier et al. (2011) located the non‐semantic structure‐building processes to the LIFG and left posterior superior temporal sulcus (LpSTS).

Timing and incremental processing can provide further evidence for a process‐based signature of hierarchical structure. To parse a sequence into a tree structure, the listener needs to place “open” terminals (those requiring additional terminal(s) to satisfy their relations) into some auxiliary memory store (e.g., a “stack” or a random access memory) until their appropriate completion terminal(s) arrive, so that they can be inserted into the nested grouping structure. Just as the presence of long‐distance dependencies is indicative of hierarchical structure, increased activation of an external memory store with increasing “open” terminals also provides a signature of hierarchical processing.

Multiple assumptions underlie this approach, for example, that all levels of nested groups that can be formed (i.e., all structures that can be built) at a certain time step will be formed at that time step. The more deeply nested a group is, the more it depends on the completion of higher nesting levels, so that it will be completed later. The number of groups that can be formed at each time step can thus be translated to a time course of nested grouping effort that can be matched to the online effort data. Examples of this approach, where the incremental dimension of hierarchical processing (of sentences) emerges, include Nelson et al. (2017) using ECoG‐data or Udden et al. (2019a), using the BOLD‐response.

To conclude this section, the list of methods in Table 1 implies multiple potential indicators of hierarchical structure, no one of which will apply in all cases or to all cognitive domains. The most convincing evidence would be cumulative, when multiple signatures are demonstrated for a particular cognitive domain (or species). In addition, “model selection” approach (Chamberlin, 1890; Platt, 1964) will be crucial for experiments attempting to distinguish sequential and hierarchical structure, since the data may be consistent with a hierarchical model, but more (or equally) consistent with a sequential one. Only when the hierarchical model clearly provides a superior fit can we confidently conclude that hierarchy is the best explanation (e.g., Ravignani, Westphal‐Fitch, Aust, Schlumpp, & Fitch, 2015; van Heijningen, de Visser, Zuidema, & ten Cate, 2009).

## 3 Neural signatures of hierarchical processing

Recall that we identified "nested grouping" as a key process by which hierarchical structure emerges (Section 2.22.3), and that additional demands on memory are a signature for hierarchical structure. Both nested grouping and such involvement of "auxiliary memory"play important roles in the literature on the neural signatures of hierarchical processing discussed next.

We will restrict our analysis to language, even though there is an interesting literature on hierarchical processing in other domains. The reason is that in those other domains, information is often not accessed via sequences with fixed order (e.g., visuospatial, social, or navigation hierarchies, in which hierarchical processing also involves nested grouping), and may therefore not involve the same auxiliary memory systems as those used to process structured sequences (as in speech or music). Moreover, two recent papers suggest specializations to particular kinds of content, even in domains where the information is presented sequentially (e.g., visual vs. verbal; Milne, Wilson, & Christiansen, 2018; Udden & Männel, 2019b).

### 3.1 Divisions of nested grouping and auxiliary memory

What memory systems are used in processing hierarchical structure? It is important to distinguish between different auxiliary memory systems, which may include activation of long‐term memory stores (e.g., lexical retrieval), or different forms of working memory (McElree, 2006). When viewing working memory capacity as distinct from long‐term memory, we should be precise regarding domain‐specificity of the memory store. Is it “just” the phonological loop (or echoic memory, or iconic memory, etc.) which is used, or do stores specific for sequence processing exist? Several recent proposals help sharpen this distinction.

Based on data from dynamic causal modeling (Makuuchi & Friederici, 2013), Friederici (2017) proposes a generic working memory system for sentence processing in inferior frontal sulcus (IFS) and the inferior parietal sulcus (IPS), connected via the superior longitudinal fasciculus, and further suggests that Merge (a process forming nested groupings) takes place in the ventral BA44, a suggestion reminiscent of Fedorenko’s proposal of a domain‐general system interacting with a neighboring domain‐specific language system. The proposed domain general system is located just dorsal to the endpoint of the arcuate fasciculus connecting LIFG and the posterior temporal lobe (Fedorenko, 2014).

In Matchin’s (2017) model, the posterior LIFG (BA45) supports syntactic working memory specifically, by applying a general working memory function to domain‐specific syntactic representations. In this model, even though this working memory function is syntax specific, it is still separated from the structure building process *per se*, suggested to rely on LpSTS and/or distributed oscillatory signals.

### 3.2 Neural signatures of nested grouping

Turning to the processes generating nested grouping *per se,* a common suggestion is that nested grouping processes may be accomplished by oscillations nested in time, a proposal complementary to the spatial localization approaches discussed above. Investigating this suggestion requires computational approaches to modelling neurophysiology, most important intrinsic neural oscillations. A recent seminal paper suggesting that nested grouping could be implemented using nested oscillations (Ding, Melloni, Zhang, Tian, & Poeppel, 2016) has inspired further suggestions integrating this hypothesis with theoretical linguistics (e.g., that brain rhythms can be naturally linked to the linguistic notion of phases; Boeckx, 2017).

In Section 2.1, we noted that the operations Merge and Unify characterize specific bottom‐up views of nested group formation. Recent work explicitly aims to localize merge, linking theoretical linguistics and neuroimaging, but results differ across two laboratories. Friederici’s group (Zaccarella & Friederici, 2015; Zaccarella, Meyer, Makuuchi, & Friederici, 2017) localized Merge to ventral BA44, in line with Friederici’s model (2017). In contrast, Sakai’s laboratory, using a parametric design with a varying number of Merge applications needed to comprehend sentences (Ohta, Fukui, & Sakai, 2013), observed increased activity along the left IFS and in the left parietal cortex with the number of merges applied. These two lines of work build on an earlier paper (Musso et al., 2003), which no longer meets today’s power standards (Button et al., 2013).

Furthermore, in a recent meta‐analysis, multiple studies including sentence versus word list conditions were reinterpreted as a merge versus no‐merge contrast (Zaccarella, Schell, & Friederici, 2017), yielding similar observations as those of Pallier et al. (2011). Although their fMRI or meta‐analytic data did not distinguish the LIFG from the pSTS, they nonetheless interpreted BA44 as the location of merge, while left pSTS was interpreted as subserving a later integration with semantics. Another recent review specifies the function of left pSTS as *labeling* (making the tree a rooted tree by categorization/headedness, cf. Goucha, Zaccarella, & Friederici, 2017).

### 3.3 Neural signatures of auxiliary memory

An ECoG‐study by Nelson et al. (2017) used a model of incremental‐generating processes where the number of open syntactic nodes varies across presented word. This was used as an explanatory variable explaining the high‐frequency component of the intracranial ECoG signal. As in the Pallier study, activity in LIFG and left pSTS corresponded well with this index of hierarchical structure generation. In a model comparison approach, their results were interpreted as supporting modeling sentences as hierarchical (rather than non‐hierarchical).

In a recent fMRI study by Udden et al. (2019a) using both visual and auditory sentence processing, these results were extended by showing a functional asymmetry in neural processing of dependencies that go from the head to the dependent (i.e., left‐branching) compared to the other way around (right‐branching). The crucial difference is that non‐attached constituents must be maintained online (e.g., pushed on a stack) only in the left‐branching case. This occurs only if there is a asymmetrical hierarchical structure present in the sentences, an analysis which the study thus supports. Parametrically increased stack depth (the number of simultaneously open left branching dependencies) correlated with activity in LIFG and left pSTS. The corresponding measure for right branching dependencies (not requiring syntactic working memory) activated the anterior temporal lobe; another complexity measure not distinguishing left from right dependencies (total dependency length) activated left pSTS only. Note, however, that both studies are still limited in the extent to which they can strictly distinguish auxiliary memory from structure building processes, as sentences with high load on memory are also structurally complex.

In order to disambiguate between these components, it can be useful to look at domains with different auxiliary memory systems. For instance, during visual‐spatial and motor hierarchical processing, neither LIFG nor pSTS seemed to support hierarchical branching (Martins et al., 2014, 2019). However, in these studies, the production of hierarchies was highly automatized. A recent voxel‐based symptom‐lesion replication with untrained participants suggests that in the visual domain, while pMTG is crucial for the acquisition of hierarchical branching rules, LIFG rather supports cognitive control.

### 3.4 Concluding observations

In summary, by gradually clarifying the central theoretical distinctions and commonalities, different authors are increasingly recognizing closely related theoretical notions of hierarchy. The empirical studies on linguistic syntax reviewed above reveal consistent activations of LIFG and left pSTS, whether they are focused on nested grouping or external memory processes (noteworthy since the studies were selected for review based on their empirical approach, not their results). Models based on the sequential/hierarchical distinction all include activation of these regions. Although some models suggest LIFG as a structure building “hotspot” and others suggest left pSTS, there is a broad consensus that sequence processing over hierarchical structures in language can be assigned to a circuit incorporating the LIFG and LpSTS, via their connections in the arcuate fasciculus.

Extending this approach beyond music and language to further cognitive domains (e.g., vision, action or spatial navigation) may help to further clarify the division of labor between brain areas. Neither spatial location nor oscillatory neural activity alone can precisely specify the computations underlying hierarchical processing. However, explicit processing models that allow stimulus material to be parameterized allow precise focus on the key hierarchy/sequence distinction. Combined with a model comparison approach, this kind of theoretical clarity will provide the necessary basis for future progress, allowing us to identify the signatures of hierarchical structure in human cognition, and further pinpoint and understand its computational and neural underpinnings.
